# Platelet-Activating Factor Receptor (PAFR) Regulates Retinal Progenitor/Stem Cells Profile in Ciliary Epithelium Cells

**DOI:** 10.3390/ijms25063084

**Published:** 2024-03-07

**Authors:** Barbara Dalmaso, Ildefonso Alves da Silva-Junior, Sonia Jancar, Carolina Beltrame Del Debbio

**Affiliations:** 1 Department of Cell and Developmental Biology, Biomedical Sciences Institute, University of Sao Paulo, Sao Paulo 05508-000, Brazil; barbdalmaso@gmail.com; 2 Department of Immunology, Biomedical Sciences Institute, University of Sao Paulo, Sao Paulo 05508-000, Brazil; ildefonsoasjr@msn.com (I.A.d.S.-J.); sojancar@icb.usp.br (S.J.)

**Keywords:** PAF receptor, Ciliary Epithelium, retinal stem cells

## Abstract

The retina is a central nervous tissue essential to visual perception and highly susceptible to environmental damage. Lower vertebrate retinas activate intrinsic regeneration mechanisms in response to retinal injury regulated by a specialized population of progenitor cells. The mammalian retina does not have populations of progenitor/stem cells available to activate regeneration, but contains a subpopulation of differentiated cells that can be reprogrammed into retinal stem cells, the ciliary epithelium (CE) cells. Despite the regenerative potential, stem cells derived from CE exhibit limited reprogramming capacity probably associated with the expression of intrinsic regulatory mechanisms. Platelet-activating factor (PAF) is a lipid mediator widely expressed in many cells and plays an important role in stem cell proliferation and differentiation. During mammalian development, PAF receptor signaling showed important effects on retinal progenitors’ cell cycle regulation and neuronal differentiation that need to be further investigated. In this study, our findings suggested a dynamic role for PAF receptor signaling in CE cells, impacting stem cell characteristics and neurosphere formation. We showed that PAF receptors and PAF-related enzymes are downregulated in retinal progenitor/stem cells derived from PE cells. Blocking PAFR activity using antagonists increased the expression of specific progenitor markers, revealing potential implications for retinal tissue development and maintenance.

## 1. Introduction

The retina is a central nervous system tissue essential for visual processing. It contains a complex arrangement of interconnected neurons that convert optical information into neural impulses. Lower vertebrates’ retinas retain intrinsic regenerative mechanisms based on the activation of local quiescent retinal stem cells (RSC), retinal progenitor cells (RPC), and ciliary marginal zone (CMZ) cells located at the retinal periphery. Injury stimuli can trigger the proliferation of RSC, RPC, and CMZ cells, which will differentiate into new retinal neurons [1].

Unfortunately, the adult mammalian retina does not have a proliferative CMZ or an RSC cell source available, resulting in an ineffective capacity to regenerate after tissue damage. However, the mammalian ciliary epithelium (CE) is a structure topologically analogous to the adult CMZ of lower vertebrates and presents a small subset of cells that exhibit promising stem-cell-like properties that could be targeted for future regenerative therapy development [2,3].

**Figure 1:**
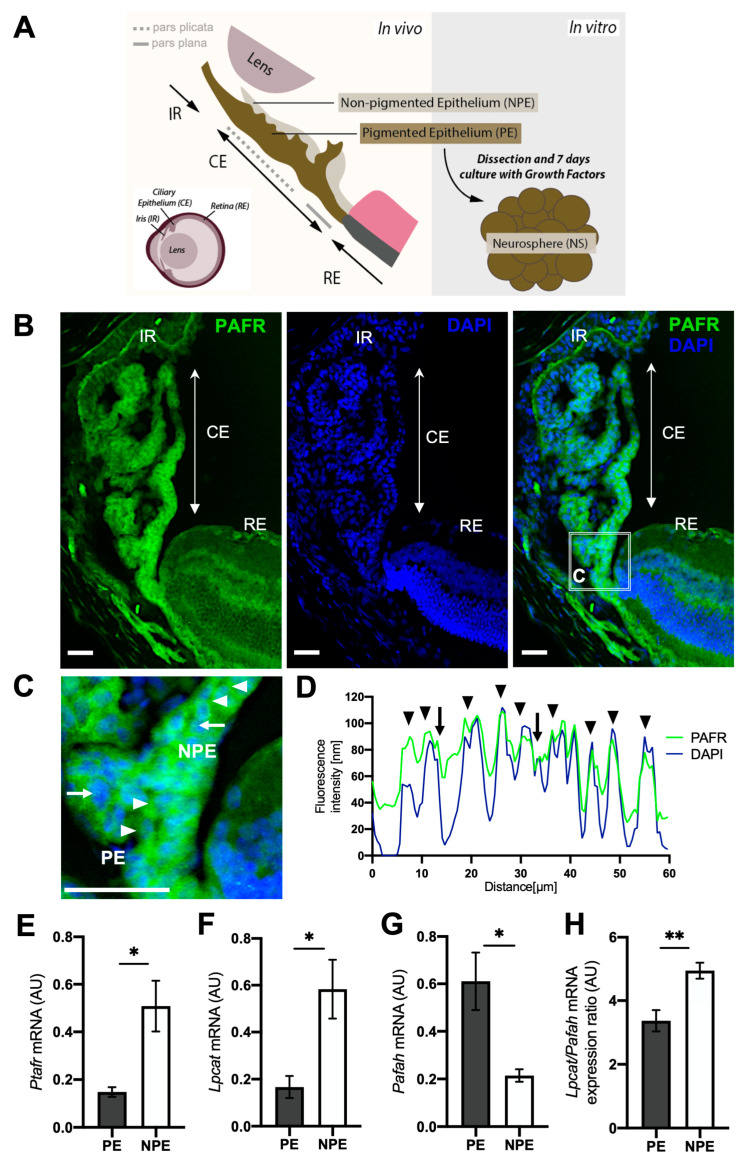
PAFR expression in CE cells. (**A**) Schematic diagram showing mouse peripheral eye anatomy and indicating the retina (RE), iris (IR), lens, and the ciliary epithelium (CE) divided into the inner non-pigmented epithelium (NPE) and the outer pigmented epithelium (PE). The PE can form neurospheres (NS) after growth factor stimulation. (**B**) Immunohistochemistry analysis of eyes’ sections showing cells positive for PAFR (green) and nuclei (DAPI) in both PE and NPE cells, as indicated by the arrowheads in the detailed image in “A” (*n* = 3). (**C**) The detailed image from figure (**B**) indicates PAFR expression in the cell membrane/cytoplasm (regular arrows) and in the nuclei (arrowheads). The white bar represents 25 μm. (**D**) PAFR and DAPI confocal fluorescence profile analysis demonstrated fluorescence expression patterns. Both fluorescence peaks colocalization (arrowhead) suggest that PAFR is present in the nuclei. The absence of colocalization peaks (regular arrow) indicates PAFR located outside the cell nuclei (green only). (**E**–**G**) Dissociated PE and NPE transcriptional expression of PAF receptor Pafr (**E**), PAF—synthesis enzyme Lpcat and (**F**) PAF—degradation enzyme Pafah (**G**). (**H**) PAF-related enzyme ratio expression, indicating higher levels of Lpcat than Pafah in NPE in comparison to PE. Results are given as mean ± S.E.M, * *p* < 0.05; ** *p* < 0.01; A.U. = arbitrary unit.

The CE is a cuboid epithelial tissue derived from the neural tube and located between the iris and retina (Figure 1A). It contains a double-layered epithelium with specialized secretory and regulatory functions, such as maintaining intraocular immune status and pressure, and aqueous humor production [4,5]. The mammalian CE contains the outer pigmented epithelium (PE), which is continuous with the retinal pigmented epithelium (RPE), and the inner non-pigmented epithelium (NPE), which is continuous with the neural retina. Depending on the species of vertebrate, the CE can be subdivided into two regions: the pars plana and the pars plicata. The pars plana is flat and is continuous with and anterior to the peripheral edge of the retina. The *pars plicata* is folded and is continuous with and anterior to the *pars plana.*

Under physiological conditions, adult mammalian CE cells have restricted proliferation potential. However, a subset of cells located at the PE layer has been reported to form neurospheres after in vitro growth factor (GF) stimuli (Figure 1A), acquiring a progenitor phenotype similar to the non-mammalian CMZ [6]. At the transcriptional level, adult PE cells exposed to GF upregulated the expression of proliferation markers (Ki67 and Cyclins) and neural progenitor genes specific to the RSC phenotype (Nestin and Pax6), while at the same time downregulating the expression of terminally differentiated cell markers, such as the epithelial gene Rab27b [7,8]. PE-derived neurospheres were capable of differentiating into different retinal neuron phenotypes and presented electrophysiological responses [2,3,9]. These results proved the existence of a subpopulation of PE cells with the ability to undergo progenitor/stem cell reprogramming, self-renewal, and generation of functional neurons upon non-cell-autonomous influences [9].

Although PE progenitor/stem cells have significant regenerative potential after GF stimulation, they fail to migrate into the neural retina in vivo to promote tissue regeneration [6]. Additionally, PE-derived neurospheres retain the expression of some epithelial and pigmentation features after stem cell reprogramming [10], limiting the neurogenesis potential.

The fact that these cells can proliferate in culture after proper stimuli suggests that intrinsic or extrinsic factors may prevent CE cells from developing full regenerative potential. The challenge now is to understand whether the cellular and molecular mechanisms to which the cells are exposed control the stem cell differentiation processes or influence the reprogramming process.

Platelet-activating factor, PAF (chemical name 1-O-alkyl-2-acetyl-sn-glycero-3-phosphocholine), is a potent bioactive phospholipid present in several cell types, such as macrophages, fibroblasts, and central nervous system tissues, including the retina [11]. Under physiological conditions, picomolar amounts of PAF are continually produced by lyso-PAF acetyltransferases (LPCATs) to maintain cell membrane composition [12,13]. After specific cell stimuli, inducible types of LPCATS activity increase, generating large amounts of PAF that act as a potent intra- and inter-cellular messenger by activating (or binding) to the PAF receptor (PAFR) [13,14]. PAF activity is downregulated by the expression of PAF acetyl-hydrolases (PAFAH), which convert PAF back to an inert form [15,16]. The intracellular PAF activity can be indirectly measured by the ratio expression of LPCAT (PAF production enzymes) and PAFAH (PAF degradation enzymes).

PAFR is an important pleiotropic G-protein-coupled membrane receptor that activates complex signaling pathways and induces intracellular calcium mobilization primarily associated with inflammation, cell cycle regulation, and differentiation [17,18]. Our group observed a positive correlation between increased PAFR expression and terminally differentiated retinal tissue. Notably, PAFR knock-out mice (PAFR−/−) displayed reduced neuronal maturation and synaptic capability compared to wild-type control animals, followed by increased proliferation markers during retinal postnatal development (unpublished data). At the transcriptome level, a separate study indicated that PAFR is down-regulated after somatic reprogramming to a stem cell state, and the absence of PAFR expression was found to be crucial in maintaining the stem cell phenotype [19]. Moreover, exogenous PAF stimulation inhibited developing RPC proliferation in vivo by arresting the cell cycle S/G2 transition [20,21]. Together, these data suggest that the PAF/PAFR pathway plays an important role in retinal progenitor cell differentiation.

Although PAFR expression was previously observed on CE cells [22], its influences on the CE progenitor profile and reprogramming have not yet been established.

## 2. Results

### 2.1. Differential Expression of PAF Receptor (PAFR) and Associated Pathway Enzymes in Ciliary Epithelium

We first characterized the expression of PAFR and PAF-synthesizing (LPCAT) and -degrading (PAFAH) enzymes in adult ciliary epithelium (CE) cells (Figure 1, Figures S1 and S2). Our immunohistochemistry analysis demonstrated the presence of PAFR-positive cells in both PE and NPE tissues with no discernible differences (Figure 1B,C, and Figure S1). The confocal fluorescence profile analysis demonstrated a high overlap between PAFR localization (green color) and the nuclear area (blue color), indicating that PAFR is detected in both the nuclear membrane (blue and green peaks colocalizing) and the cell membrane (blue and green peaks that do not colocalize) (Figure 1D). The quantitative transcriptional analysis of dissociated CE cells indicated higher levels of PAF receptor (Ptafr) in NPE in comparison to PE (Figure 1E). The PAF-synthesizing enzyme (Lpcat) was also up-regulated in NPE cells in comparison to PE (Figure 1F), and the PAF-degrading enzyme (Pafah) was downregulated in the NPE tissue (Figure 1G). To indirectly determine the activity levels of PAF in each cell type, we calculated the ratio expression between the PAF production and degradation enzymes (Lpcat2/Pafah, respectively) in these cells. Our analysis indicated a higher ratio of Lpcat2/Pafah in NPE tissue (Figure 1H), suggesting that Lpcat expression overcomes the expression of Pafah in NPE.

### 2.2. PAFR Expression in Quiescent Retinal Progenitor/Stem Cells

It is known that a subset of PE cells from mammalian CE retains quiescent RPC properties, demonstrated by the ability to form neurospheres in vitro containing a population of heterogeneous progenitor cells. The NPE cells, on the other hand, lack this feature and fail to form neurospheres in the presence of growth factors. Our results demonstrate that PAF activity is upregulated in NPE in comparison to PE. Based on these results, we question if the lower levels of PAFR and PAF-related enzymes could be related to higher retinal/progenitor stem cell features. We investigated this hypothesis in two different ways: first, we analyzed the PAF signaling in two different populations of retinal stem cells from zebrafish, one with higher potential (RSC) and the other with limited potential (CMZ). Later, we analyzed the expression of retinal progenitor markers in PE cells derived from PAFR knockout (PAFR−/−) animals in comparison to wild types.

**Figure 2:**
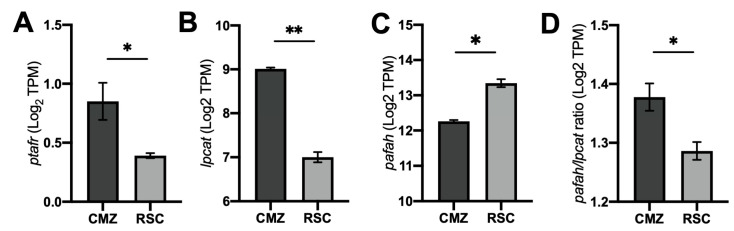
Bioinformatic analysis on gene expression of zebrafish-derived retinal stem cells (RSC). (**A**) The quantitative transcriptional analysis indicated higher PAFR (ptafr) expression in quiescent adult progenitor cells in the CMZ (*n* = 3) compared to activated developing RSC (*n* = 4). (**B**,**C**) The quantitative transcriptional analysis indicated increased PAF-synthesis enzyme lpcat (**B**) and decreased PAF-degradation enzyme pafah (**C**) in CMZ cells. (**D**) The CMZ presented a higher transcriptional ratio of pafah/lpcat2, suggesting enhanced PAF production on differentiated tissue. CMZ = Ciliary marginal zone. RSC = Retinal stem cells. Results are given as mean ± SEM of gene counts after log2 normalization. * *p* < 0.05, ** *p* < 0.01. Retrieved scRNA-seq datasets from Xu et al., 2020 [23].

First, we retrieved zebrafish RNA-seq datasets from embryonic retinas (containing a population of retinal stem cells (RSC) with great potential to differentiate into several cell types) and from adult CMZ cells (containing a population of limited-fate quiescent adult progenitor cells). We observed that adult CMZ expressed higher levels of PAFR (ptafr) in comparison to RSC (Figure 2A). The upregulation of ptafr in CMZ was followed by increased lpcat and decreased pafah expression (Figure 2B,C). A higher expression ratio of lpcat/pafah was also found in CMZ tissue (Figure 2D), suggesting that lpcat expression overcomes the expression of pafah in more differentiated zebrafish retinal stem cells.

**Figure 3:**
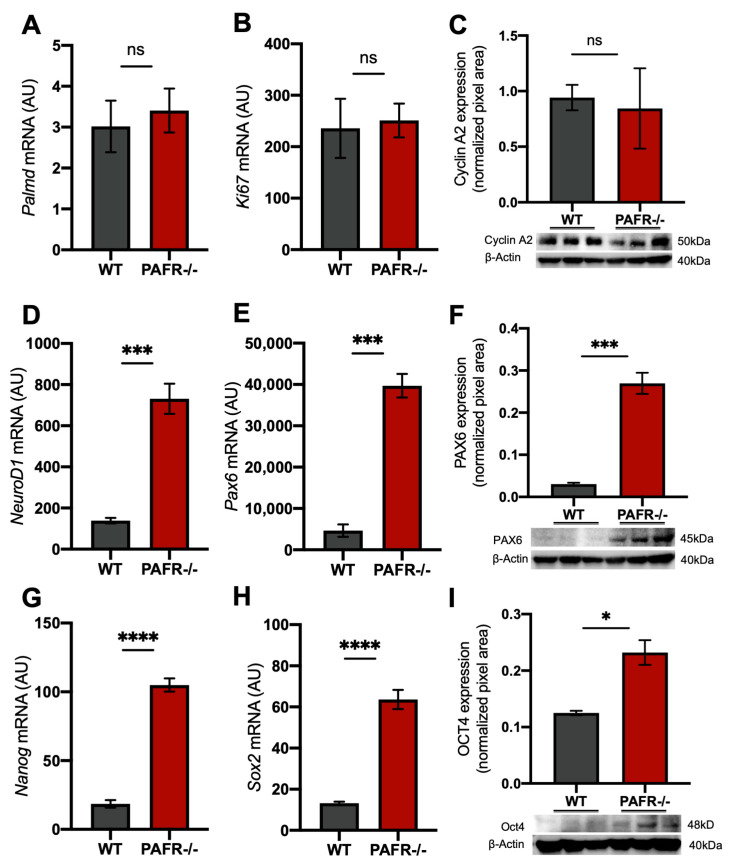
Progenitor/pluripotent markers expression on the pigmented epithelium (PE) of PAFR null mice (PAFR−/−). (**A**,**B**) The expression of CE differentiation (Palmd) and cell proliferation (Ki67) transcripts. (**C**) The total protein expression of cell cycle marker Cyclin A2. (**D**) The transcriptional expression of neural marker NeuroD1. (**E**,**F**) Retinal progenitor marker Pax6 transcripts and protein expression. (**G**–**I**) Pluripotent markers Nanog, Sox2, and Oct4 expression. Results are given as mean ± SEM. (*n* = 4). * *p* < 0.05, *** *p* < 0.0005; **** *p* < 0.0001; ns = non-significant. A.U. = arbitrary unit.

Next, we evaluated the transcriptional expression of different retinal progenitor markers in the PE cells from PAFR−/− mice. We confirmed that PAFR was absent in the CE cells from PAFR−/− mice via immunohistochemistry (Supplementary Figure S3). The transcriptional analysis revealed no differences in the expression of differentiated PE marker (Palmd) and cell cycle marker (Ki67) between wild-type and PAFR−/− PE cells (Figure 3A,B). No differences in cyclin A2 total protein were detected (Figure 3C). Surprisingly, significantly higher levels of specific neuronal and retinal progenitor markers (NeuroD1 and Pax6) (Figure 3D–F) and pluripotent markers (Nanog, Sox2, and Oct4) (Figure 3G–I) were detected in the PE of PAFR−/− mice.

Together, these results suggest that PAF signaling is downregulated in retinal stem cells and could influence the regulation of retinal progenitor markers and stem cell characteristics.

### 2.3. PAFR and PAF-Related Enzyme Expression Is Similar in PE and PE-Derived Neurospheres

**Figure 4:**
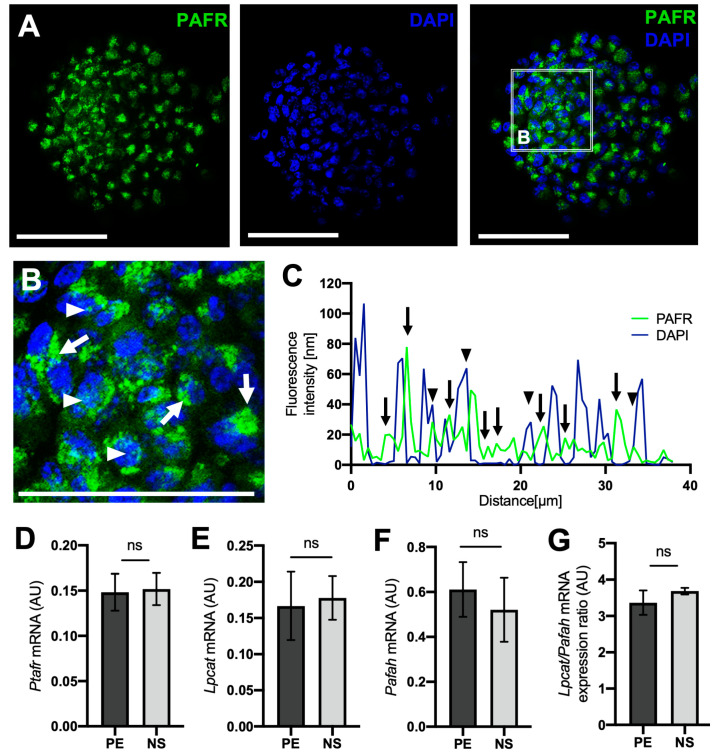
PAF receptor and PAF-related enzyme expression analysis on PE cells and neurospheres. (**A**) Immunohistochemistry analysis of neurospheres for PAFR (green), cell nuclei (DAPI, blue), and the merged image (*n* = 3). (**B**) Detailed image from the figure (**A**) indicating PAFR expression in the cell membrane/cytoplasm (regular arrows) and in the nuclei (arrowheads). The white bar represents 25 μm. (**C**) Confocal fluorescence profile analysis indicating lower rates of colocalization peaks, suggesting that PAFR is located outside the cell nuclei (green only). (**D**–**G**) PAFR, PAF-related enzymes, and LPCAT2/PAFAH ratio mRNA expression in the pigmented epithelium (EP) and in PE-derived neurospheres (NS). Data are given as mean ± SEM of the comparative delta-CT (*n* = 4). ns = non-significant. A.U. = arbitrary unit.

Isolated PE cells from control animals were cultivated with growth factors to activate the quiescent progenitor cells and induce neurosphere formation. Most of the cells within the neurospheres (NS) expressed PAFR (Figure 4A). The confocal fluorescence profile analysis indicated that PAFR expression was mainly located in the cell membrane and not in the nucleus (Figure 4B,C). Transcriptional analysis of Ptafr indicated no differences in PAFR expression between neurospheres and its original cell source, the PE (Figure 4D). Similarly, Lpcat2, Pafah, and the Lpcat2/Pafah ratio presented no differential expression (Figure 4E–G), suggesting that PE-derived neurospheres maintain similar levels of PAF signaling and activity profile from the original tissue (PE).

### 2.4. PAFR Pharmacological Modulation-Regulated Neurosphere Formation and Retinal Stem Cell Marker Expression

To understand the role of PAFR in RPC from neurospheres, we cultured PE cells with a PAFR antagonist (PCA4248) or the agonist (cPAF) during progenitor cell reprogramming. First, a toxicity assay was performed to determine the safe doses of both drugs to be used in vitro (calcein-AM assay). A threshold of a minimum of 70% viable cells was considered appropriate (Supplementary Figure S4A,B). Then, we indirectly investigated the physiological effect of the drugs by measuring the concentration of the calcium-binding protein CaM mRNA (Supplementary Figure S4C–E). The activation of PAFR is known to increase intracellular calcium (Ca), inducing the upregulation of CaM expression. Based on cell viability results and PAFR activity, we selected the dose concentrations of 10 µM for PCA4248 and 100 nM for cPAF treatment.

**Figure 5:**
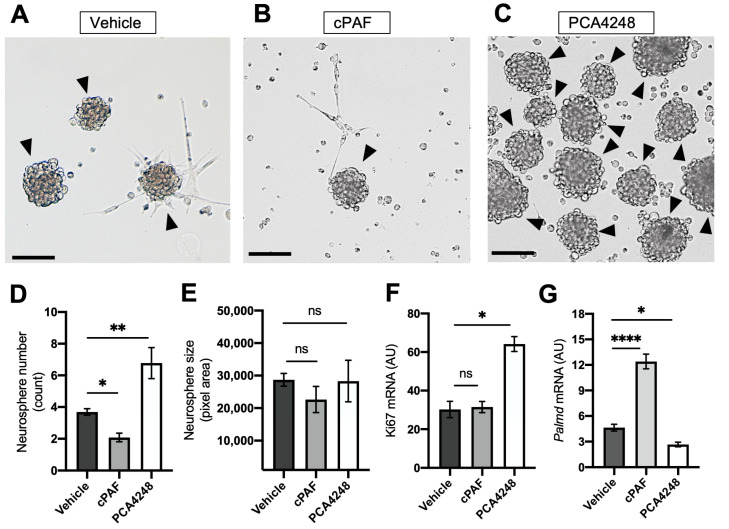
PAFR agonist and antagonist effect on pigmented epithelium (PE) neurosphere formation. Optical microscopy analysis after 7 days of treatment with (**A**) control vehicle (1 μM DMSO), (**B**) 100 nM of PAFR agonist (cPAF), or (**C**) 10 μM of PAFR antagonist (PCA4248). Black arrowheads indicate neurospheres and the scale bar represents 25 μm. (**D**) Neurosphere counting analysis. (**E**) Neurosphere size measured in pixel density. (**F**) Transcriptional expression of proliferation marker ki67. (**G**) Transcriptional expression of the ciliary epithelium marker Palmd. Results are given as mean ± SEM, * *p* < 0.05, ** *p* < 0.01, **** *p* < 0.0001, ns = non-significant (*n* = 4). A.U. = arbitrary unit.

PE cells were treated for 7 days with PCA4248, cPAF, or vehicle (DMSO) during neurosphere formation (Figure 5). We first checked the presence of neurospheres, neurosphere size, and quantity after 7 days in culture. Considering that each neurosphere originated from a single progenitor cell with the ability to proliferate, the neurosphere size depends on the cell cycle reentry rates of this progenitor cell, suggesting that larger neurospheres present higher proliferation rates. On the other hand, the quantity of neurospheres in culture reflects the amount of progenitor cells present in the PE tissue. This means that the number of neurospheres generated in culture reflected the number of progenitor cells present in the PE tissue collected from the animal, the more neurospheres, the more RPC and vice versa.

The treatment with PAFR agonist cPAF decreased the PE cell’s ability to form neurospheres in culture, significantly changing the number of formed neurospheres (Figure 5A,B,D). On the other hand, the treatment with PAFR antagonist PCA4248 significantly increased the number of neurospheres in culture (Figure 5C,D). Neither cPAF nor PCA4248 changed neurospheres’ size in comparison to controls (Figure 5E). Interestingly, neurospheres treated with PCA4248 presented higher levels of Ki67 transcripts (Figure 5F), which could be related to the higher number of neurospheres formed in culture rather than to the cell cycle re-entry. This hypothesis must be confirmed.

The transcriptional expression of Palmd was increased in neurospheres treated with cPAF in comparison to controls and PCA4248 groups (Figure 5G), suggesting a higher number of differentiated cells after PAFR activation. PCA4248 treatment induced the opposite effect, decreasing Palmd expression in neurospheres in comparison to controls (Figure 5G).

**Figure 6:**
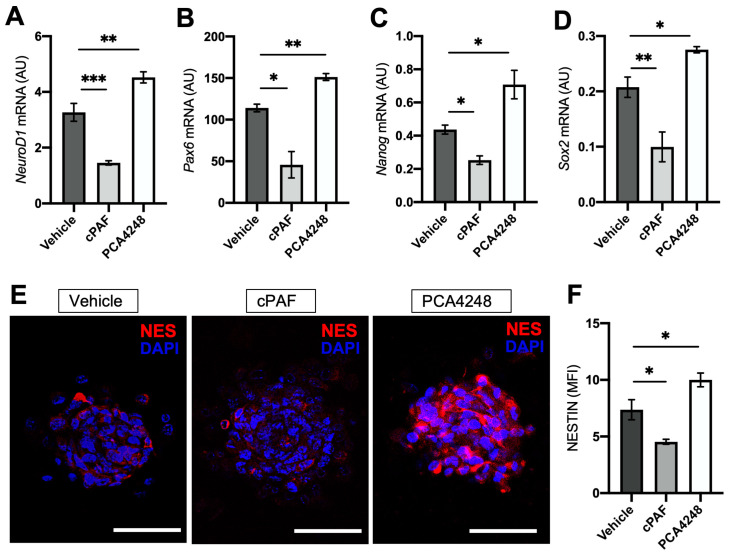
PAFR agonist and antagonist’s effect on progenitor/stem cell markers. (**A**–**D**) mRNA expression of progenitor and pluripotent markers after 7 days of treatment with control vehicle (1 μM DMSO), 100 nM of PAFR agonist (cPAF), or 10 μM of PAFR antagonist (PCA4248). The white bar represents 25 µm (*n* = 4). (**E**) Immunohistochemistry analysis on nestin expression (neural progenitor marker) and cell nucleus (DAPI) (*n* = 3). (**F**) Mean fluorescence intensity (MFI) analysis for NESTIN. Results are given as the mean ± SEM of comparative delta-CT. * *p* < 0.05, ** *p* < 0.01, *** *p* < 0.001, ns = non-significant. A.U. = arbitrary unit. Scale bar = 25 µm.

We also investigated the influence of PAFR pharmacological modulation on different retinal progenitor/stem cell marker expression. As expected, PAFR agonist treatment downregulated the transcriptional expression of the neural progenitor marker NeuroD1 (Figure 6A), RPC marker Pax6 (Figure 6B), and pluripotent markers Nanog and Sox2 (Figure 6C,D). Interestingly, PAFR antagonist treatment induced the opposite effect on PE neurospheres, significantly increasing the transcriptional expression of the same markers in comparison to controls.

Immunohistochemistry analysis indicated a decrease in the expression of the neural progenitor marker Nestin after PAFR agonist treatment, but an increase in protein expression after antagonist treatment (Figure 6E,F). Together, these results demonstrate that PAFR blockage during neurosphere development can promote the transcriptional expression of progenitor markers and that exogenous agonist treatment can inhibit this process.

## 3. Discussion

Retinal degenerative diseases and blindness pose a global threat to public health. Retinal degeneration is an irreversible condition underlying visual impairment and blindness, significantly reducing the patient’s quality of life. With no efficient treatment currently available for retinal degeneration and gene therapy still under development, studies aiming to replace damaged cells or tissue through cell therapy hold great clinical potential [24].

Various types of stem cells have undergone testing in both preclinical and clinical trials to potentially reverse retinal degeneration [25,26,27]. While promising results have already been achieved, demonstrating the significant regenerative potential of stem cells, there are still critical aspects that demand attention. Some clinical and preclinical studies have reported adverse effects related to transplantation, including the potential for tumor formation from residual undifferentiated cells. As of now, data on the long-term safety of highly potent cells derived from human embryonic stem cells (hESCs) and induced pluripotent stem cells (iPSCs), particularly regarding teratoma formation after transplantation, remain unavailable [25,27].

Stem cells can be classified based on their potency, which is associated with their ability to differentiate into multiple cell types. Progenitors or multipotent stem cells have limited potential for differentiation according to the tissue of origin, while pluripotent stem cells have the capacity to differentiate into any cell type found in the adult body. Despite the considerable regenerative potential of pluripotent stem cells, strategies centered on stem cell-based approaches that aim to replace endogenous cells by activating stem-cell-like populations within the diseased retina represent a safer approach [28].

In this context, mammalian retinal progenitor cells derived from the pigmented epithelium (PE) of the ciliary body have emerged as a promising cell source for cell replacement therapy. This is primarily due to their analogous characteristics to the ciliary marginal zone (CMZ), a crucial tissue for the retinal regeneration observed in lower vertebrates. Despite these promising features, there are still gaps in our understanding of the factors and pathways that control their ability to regenerate retinal tissue and their potential for cell differentiation, necessitating further investigation. Here, we present evidence that the platelet-activating factor (PAF) receptor plays a significant role in ciliary epithelium (CE) stem cell reprogramming and the maintenance of a progenitor profile. This finding could contribute as another piece of the puzzle in comprehending the regenerative potential of these cells.

We demonstrated that the PAF receptor (PAFR) and enzymes related to the PAF pathway are expressed in CE cells, with lower levels detected in pigmented epithelium (PE) cells (considered as retinal progenitor cells), in comparison to non-pigmented epithelium (NPE) cells (which fail to reprogram into retinal progenitor cells and are not considered as retinal stem cells).

Wang et al. investigated PAF signaling in a spinal cord injury model and observed that disrupting this pathway, either through PAFR deletion or antagonist administration, regulated the activation of microglia and astrocytes, leading to extracellular matrix remodeling. This disruption also promoted axonal regeneration and functional recovery [29]. The authors reported that blocking PAF signaling attenuated the effects of reactive gliosis and, to some extent, promoted axonal plasticity/regeneration and functional recovery. This suggests that PAF likely plays a role in modulating both the inflammatory response and neural protection [29].

The LIS1 protein, a regulatory subunit of the catalytic enzyme PAF-AH, genetically interacts with progenitor genes Sox2 and Sca-1, and the neurodevelopmental protein Nde1 [30,31,32,33]. The synergism of LIS1 with progenitor mediators is essential for stemness maintenance. The suppression of LIS1/SOX2, LIS1/Sca-1, or LIS1/Nde1 complex interactions disrupts stem cell functions, leading to decreased self-renewal, migration, and cell proliferation [33,34,35]. Neural progenitors of LIS1+/− mice show impaired responses to growth factor signals (EGF and FGF), resulting in premature differentiation and a lack of cell proliferation [33].

It has also been reported that ovarian cancer cell lines treated with PAF increase spheroid formation, promote ovarian cancer progression, induce chemoresistance, and significantly upregulate progenitor gene expression via PAF/PAFR-mediated inflammatory signaling pathways [36].

The multifaceted effects of PAF and PAFR signaling in different cell types and tissues should be interpreted carefully. PAF is strongly associated with inflammatory responses, and many experiments investigating the role of PAF use tissue injury models or tumors, make it challenging to evaluate the effect of PAF separately from the system’s inflammatory reaction.

To avoid the influence of external factors on cellular responses, we dissected the PE from NPE cells and reprogrammed the cells to become retinal progenitor cells (RPC) in culture. We observed that PE cells treated with PAFR agonists during the reprogramming stage increased the differentiated epithelial transcriptional phenotype (Palmd) and reduced the expression of progenitor markers (NeuroD1, Pax6, Nanog, and Sox2). In contrast, PAFR inhibition yielded the opposite effect, inducing PE reprogramming into neurospheres and enhancing its progenitor phenotype. Surprisingly, no discernible differences in neurosphere size were observed following PAFR pharmacological manipulation. This suggests a direct influence of PAFR on the progenitor phenotype but not a robust regulation of cell proliferation, as observed in other retinal stem cell experimental models [21].

Evidence has demonstrated that PAF/PAFR may play an important role in stem cell profile and maintenance. In the retina, it is known that PAF activation arrests retinal developing neuroblasts in vivo, leading to an unbalanced S/G1 cell cycle phase transition and decreased cyclin B1 expression [21]. Furthermore, PAFR appears to orchestrate receptor transactivation and regulates the expression of important growth factors, including EGF and VEGF [37,38]. Kume and Shimizu demonstrated that rat kidney fibroblast cells (NRK) treated with C-PAF (PAF analog) and a PAFR agonist completely suppressed the EGF-induced mitogenic response during G1-phase, suggesting an inhibitory effect of PAF on the EGF signaling pathway [39]. It is well known that EGF, together with FGF, are crucial for CE cell reprogramming, stimulating the cell cycle progression and the expression of retinal progenitor cell genes [2,40].

PAF has also been reported to stimulate the activation of the Rho GTPases family of proteins, specifically Rac1 and RhoA/ROCK [41,42]. In a previous study published by our group, it was observed that the in vivo activation of Rho GTPases substantially enhanced CE cells’ co-expression of pivotal progenitor transcription factors, such as Pax6 and Chx10 [43]. Intriguingly, Rho GTPase inactivation increased cell proliferation and potentiated the proliferative effect of growth factors [43]. This information suggests that PAF might interact with different growth factor pathways and regulate progenitor cell proliferation and differentiation.

While the precise roles of PAF/PAFR in retinal progenitor/stem cell maintenance and cell fate determination are being elucidated, their involvement in several retinal pathologies has already been described in the literature [44,45]. A recent review on the involvement of PAF/PAFR in ocular pathologies was published by our group. Briefly, elevated PAF levels are detected in response to diverse retinal pathologies involving inflammation and cellular damage. Animal models of retinal dystrophy (rd11 and B6-JR2845) revealed that LPCAT1 was responsible for maintaining specific cell membrane lipidic composition and retinal homeostasis during the degeneration process. A cross-sectional randomized study demonstrated that PAF levels were reduced in the serum of patients with Age Macular Degeneration. Animal models of induced uveitis reduced the inflammation in the ciliary body and iris area after treatment with PAFR antagonist (SRI 63-441), reversing the uveitis condition. Injured corneal cells induced the accumulation of PAF and the upregulation of PAFR expression, promoting PAFR activation after corneal injury. The proteomic profile of diabetic rat retina demonstrated increased expression of PAF-AH when compared to non-diabetic animals.

Interestingly, recent investigations have underscored the critical role of inflammation in the reprogramming of multipotent retinal stem cells during regenerative processes. Prolonged inflammatory responses mediated by retinal microglia/macrophages have been shown to impact the proliferation of RPCs and impede the induction of photoreceptors’ regenerative response [46,47,48]. This implies that a nuanced inflammatory milieu is conducive to cellular reprogramming and cell cycle re-entry, whereas excessive inflammation may exert inhibitory effects on retinal regeneration [49]. Intriguingly, mitigation of excessive inflammation through dexamethasone intervention has demonstrated in vivo restoration of RPCs regenerative phenotypes [46]. Likewise, in vitro treatment with dexamethasone has also exhibited an augmentation in the self-renewal and proliferation of CE neurospheres [50].

The precise signaling pathways involving PAF and the regenerative potential of retinal RPCs remain elusive and warrant further in-depth investigations. However, the multifaceted relationship involving PAF, inflammation, and regenerative prowess not only unravels the complexity of retinal regeneration but also opens doors to possible therapeutic interventions capable of finely tuning the cellular microenvironment for optimized regenerative responses in mammals.

In conclusion, we suggest that PAF could be an important regulator for progenitor cells derived from the CE, as PAFR activation stimulates the differentiated phenotype and PAFR inhibition increases its progenitor profile. Pharmacological manipulation of PAFR activity could improve stem cell maintenance or cell differentiation, depending on the desired outcome. Thus, PAFR regulation of CE differentiation and stem cell progenitor phenotype can be a promising target for stem cell enhancement or a pharmacological adjuvant in future therapeutic approaches.

## 4. Materials and Methods

### 4.1. Animals

Adult BALB/cJ wild-type (WT) and BALB/cJ PAFR knock-out (PAFR−/−) mice were housed in the Department of Cell and Developmental Biology’s Animal Facility at the University of Sao Paulo, Brazil. The animals were maintained on a 12 h light/dark cycle, with access to water and food ad libitum. All experiments were conducted following the guidelines adopted by the Brazilian Society of Sciences in Laboratory Animals (SBCAL) and approved by the Ethical Committee for Animal Research of the Institute of Biomedical Sciences of the University of Sao Paulo (protocol number #3588090419).

### 4.2. CE Tissue Dissection and Neurosphere Formation Assay

Adult BALB/cJ mouse eyes were enucleated, and the ciliary epithelium (CE) was dissected using an Olympus SZ40 stereoscope (Center Valley, PA, USA). Briefly, the pigmented epithelium (PE) was manually isolated from the non-pigmented epithelium (NPE) in a sterile HBSS medium. For mRNA analysis, PE and NPE were directly frozen and later processed. For neurosphere formation, dissected PE was incubated with 0.25% trypsin, 1 mM EDTA, and 1 mg/mL DNAse solution at 37 °C for 35 min. Then, dissociated PE cells were cultivated with DMEM/F12 (Dulbecco’s Modified Eagle’s Medium) enriched with N2 supplement, L-glutamine (2 mM), 1% solution of penicillin and streptomycin (10,000 Units/mL and 10,000 μg/mL), and growth factors FGF2 (10 ng/mL) and EGF (10 ng/mL) at 37 °C under a humidified atmosphere containing 5% CO_2_. Neurospheres were collected on day 7. To evaluate the influence of PAF signaling, PE cells were treated with either 100 nM of agonist cPAF (1-hexadecyl-2-N-methylcarbamyl glycerophosphocholine—carbamyl-PAF) or 10 μM of PAF receptor antagonist PCA4248 (Tocris Bioscience, Bristol, UK) every 48 h during neurosphere formation. After 7 days, neurospheres were collected and processed for mRNA analysis or fixed with 4% PFA for immunofluorescence assay.

### 4.3. mRNA Analysis

Total RNA was isolated using the RNeasy kit (Qiagen, Hilden, Germany), and cDNA (1 μg) was synthesized using SuperScript™ Reverse Transcriptase reagent (Thermo Fisher Scientific, Cambridge, MA, USA). Specific transcripts were amplified with gene-specific forward and reverse primers (Table 1) using the Quantifast SYBR Green PCR Kit (Qiagen, Hilden, Germany) on a QuantStudio 3 PCR System (Thermo Fisher Scientific, Cambridge, MA, USA). Normalized transcriptional expression was calculated as previously described, applying the Q-Gene Software method (v1.0) [51], with β-Actin as a housekeeping gene.

### 4.4. PAFR Agonist and Antagonist Treatment and Cytotoxicity Assay

Dissociated PE cells were plated in culture medium and treated with increased concentrations of the PAFR agonist cPAF (1 nM, 10 nM, 100 nM, and 1000 nM) or PAFR antagonist PCA4248 (1 μM, 3 μM, 10 μM, and 30 μM). Control groups were treated with DMSO 1 μM (vehicle). After 48 h of incubation, the cells were washed, and the Calcein-AM probe was added to the plate to perform a cytotoxicity assay (Sigma, Darmstadt, Germany). After a 30 min incubation, cell fluorescence was measured in a spectrophotometer at 485/530 nm, where the fluorescence intensity measurement was proportional to the number of viable cells.

### 4.5. Immunofluorescence

Enucleated eyes were fixed with 4% paraformaldehyde (PFA) for 30 min at room temperature and transferred to a 30% sucrose solution in phosphate buffer (PB) for 24 h at 4 °C. Cryoprotected eyes were embedded in OCT solution and cryostat-sectioned. Neurospheres were plated on poly-D-lysine-coated glass coverslips for 6 h and then fixed with 4% PFA for 15 min. Both tissue sections and neurospheres were incubated with a non-specific site-blocking solution (1% bovine albumin, 10% animal serum, and 0.3% PB/Triton X-100) for 60 min and then incubated with specific primary antibodies at room temperature overnight. The primary antibodies used in this study include rabbit anti-PAFR at 1:100 dilution (Cayman Chemical, Ann Arbor, MI, USA) and mouse anti-Nestin at 1:200 dilution (Sigma Aldrich, Burlington, MA, USA). After incubation, samples were washed in buffer solution and incubated for 2 h at room temperature with specific secondary antibodies (1:500, Alexa Fluor 488 or 555, Thermo Fisher), and DAPI at 1:1000 dilution. Images were obtained using a Zeiss LSM 800 confocal microscope and analyzed using ImageJ software (v1.54c). Manipulation of the images was restricted to threshold and brightness adjustments to the whole image. Controls for the experiments consisted of the omission of primary antibodies, and no staining was observed in these cases.

### 4.6. Western Blot (WB)

Samples were immersed in ice-cold 20 mM Tris/HCl (pH 8.0), with protease inhibitors (0.4 mM phenylmethylsulfonyl fluoride, 20 μM leupeptin, 0.005 trypsin-inhibiting U/mL aprotinin, 2 μg/mL soybean trypsin inhibitor), and homogenized. Cell debris was discarded after centrifugation (15,000× *g* for 15 min at 4 °C), and protein quantification was determined via the Bradford assay (Bio-Rad Laboratories, Boston, MA, USA). Thirty micrograms of protein per sample were separated by electrophoresis on a 12% SDS-PAGE gel and transferred to nitrocellulose membranes. Non-specific binding sites were blocked with 5% bovine serum albumin (BSA) in TBS-T buffer (150 mM NaCl, 20 mM Tris, 0.5% Tween 20, pH 7.4) for 1 hour. After washing with TBS-T, the membranes were incubated overnight with primary antibodies against Cyclin A2 (1:1000) (Thermo Scientific), Pax6 (1:500) (Thermo Scientific), Oct4 (1:500) (Thermo Scientific), or β-actin (1:1000) (Cell Signaling) at 4 °C. After the overnight incubation, membranes were washed and incubated for 2 h at room temperature with specific peroxidase-conjugated secondary antibodies (1:2000). Detection of proteins was achieved using SuperSignal West Pico Chemiluminescent Substrate (Thermo Fisher Scientific, MA, USA) and obtained with the imaging system GBox Chemi XX6 and GeneSys Software (v2.3.1) (Syngene, Bengaluru, India). Densitometric analyses were performed using ImageJ imaging software and represented as arbitrary units (A.U.) relative to β-actin quantification. The original images of WB membranes can be accessed in Supplementary Figure S5.

### 4.7. Gene Expression Datasets

Danio rerio (Zebrafish) single-cell RNA-seq (scRNA-seq) FASTQ transcriptome data were retrieved from GSE122680 [23] and analyzed using the Galaxy v2.11.0 environment [52,53]. Ciliary marginal zone (CMZ) datasets were collected from 14 day-post-fertilization (dpf) retinas (GSM3478019 and GSM3478016). Retinal stem cell (RSC) datasets were collected from 24-, 36-, and 48-h-post-fertilization (hpf) retinas (GSM3478013, GSM3478014, GSM3478015, and GSM4233148). Transcriptome scRNA-seq datasets were trimmed (TrimGalore, v0.4.4), and the sequence quality was validated (FastQC, v0.11.5). Reads were aligned to the GRCz11 reference genome, and known transcripts were quantified (Bowtie2, v2.4.2) [54]. Differentially expressed genes were obtained (FeatureCounts, v2.0.1, and Deseq2 v2.11.4) [55,56] as gene counts and then normalized in log2. From these datasets, we analyzed the expression of ptafr, lpcat2, and pafah genes in CMZ and RSC.

### 4.8. Samples and Statistical Analysis

We manually dissected the pigmented epithelium (PE) and non-pigmented epithelium (NPE). For each cell type, we pooled the samples collected from 7–10 different animals and homogenized them. Each pool of epithelial cells was considered as 1 independent sample. We analyzed 3–4 independent samples (N = 3 or N = 4) per experiment.

For neurosphere immunofluorescence experiments and neurosphere reprogramming analysis, 5 random cultured areas of each experiment were analyzed. We compared the mean fluorescence intensity (MFI) of positive cells from different experimental conditions from 3 independent experimental replicates. For CE immunofluorescence analysis, 10 sections per eye from 3 different animals were analyzed. For gene expression datasets, normalized data were compared using an unpaired *t*-test. All statistical analyses were performed using GraphPad Prism Software (v8.0), and statistical differences were determined between treatments and respective controls using an unpaired Student’s t-test (two-tailed) or one-way ANOVA. Results are expressed as mean ± SEM (standard error of the mean), and a threshold of *p* < 0.05 was used for each test.

## Figures and Tables

**Table 1:** List of specific primers.

Gene	Sequence	Accession Number
*Ptafr*	F: 5′-AGCAGAGTTGGGCTACCAGA-3′ R: 5′-TGCGCATGCTGTAAAACTTC-3′	NM_001081211.2
*Lpcat2*	F: 5′-CCAGGTGGCATTTAAGCTCT-3′ R: 5′-TCTTGGCATATTCTGGGTGC-3′	NM_173014.2 NM_001357375.1 NM_001357374.1
*Pafah*	F: 5′-GTCTCTGCTTCAGAGGATGC-3′ R: 5′-ACATTGTGATCGTGACCGTG-3′	NM_013625.4
*Palmd*	F: 5′-ATTCTCTTCCTCTCTCCCTGCTGC-3′ R: 5′-GCTACCATAAATCAAGGTGCGTCC-3′	NM_023245.3
*Ki67*	F: 5′-TAACGCCACCGAGGACAAAT-3′ R: 5′-TTCAGAAGCTCCACTTCGCC-3′	NM_001081117.2
*NeuroD1*	F: 5′- ACGCAGAAGGCAAGGTGTCC -3′ R: 5′- TTGGTCATGTTTCCACTTCC -3′	NM_010894.3
*Pax6*	F: 5′-TCTGGGCAGGTATTACGAGAC-3′ R: 5′-TTATCGTTGGTACAGACCCCCT-3′	NM_001310146.1 NM_001310145.1 NM_001310144.1 NM_001244202.2 NM_001244201.2 NM_013627.6 NM_001244200.2 NM_001244198.2
*Nanog*	F: 5′-CACAGTTTGCCTAGTTCTGAGG-3′ R: 5′-GCAAGAATAGTTCTCGGGATGAA-3′	NM_028016.3 NM_001289828.1 NM_001289831.1 NM_001289830.1
*Sox2*	F: 5′-GCGGAGTGGAAACTTTTGTCC-3′ R: 5′-GGGAAGCGTGTACTTATCCTTCT-3′	NM_011443.4
*CaM*	F: 5′-GTCATGCGGTCACTGGGTCAG-3′ R: 5′-CAAGAACTCTGGGAAGTCAATGG-3′	NM_009790.5 NM_001313934.1
*β-actin*	F: 5′-TGAGCTGCGTTTTACACCCT-3′ R: 5′-GCCTTCACCGTTCCAGTTTT-3′	NM_007393.5

## Data Availability

Data contained within the article.

## Supplementary Materials

The following supporting information can be downloaded at: https://www.mdpi.com/article/10.3390/ijms25063084/s1.
