# Supplementary material for: Platelet-Activating Factor Receptor (PAFR) Regulates Retinal Progenitor/Stem Cells Profile in Ciliary Epithelium Cells
Source: Int J Mol Sci. 2024 Mar 7;25(6):3084. doi: 10.3390/ijms25063084 (PMC10970094; doi:10.3390/ijms25063084)
Supplement: Supplementary file 1 [file ijms-25-03084-s001.zip › ijms-2827348-supplementary.pdf]

International Journal of
Molecular Sciences

MDPI

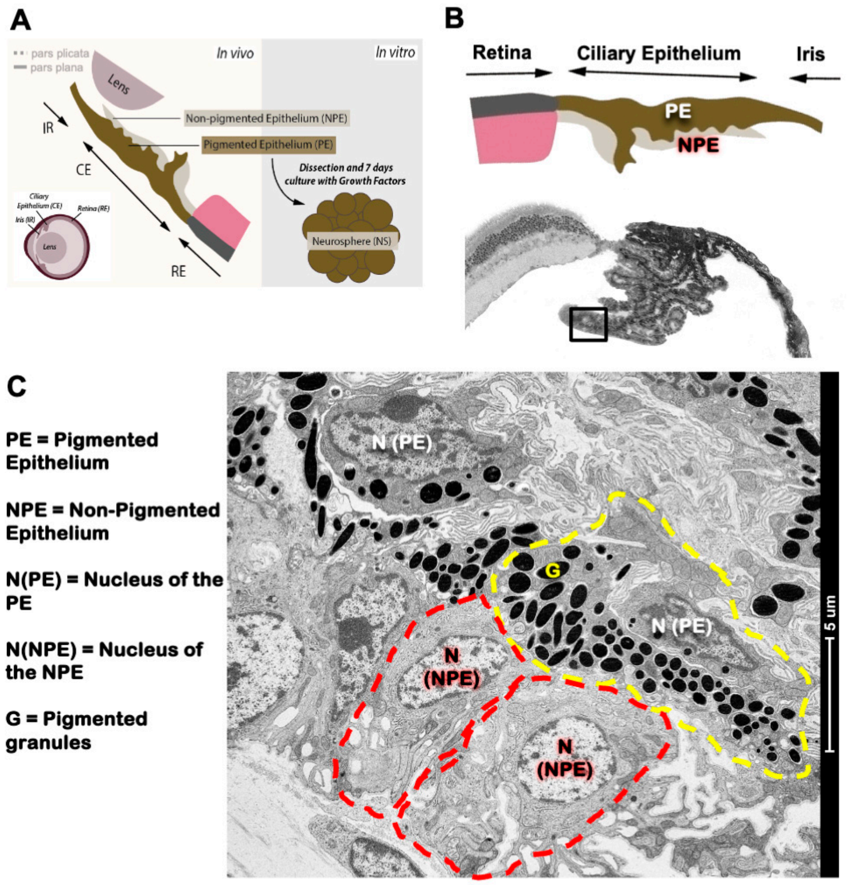

Figure S1. Ciliary Epithelium structure. (A) Schematic diagram of the Mouse peripheral eye anatomy and indicating the Retina (RE), Iris (IR), Lens, and the Ciliary Epithelium (CE) divided into the inner Non-Pigmented Epithelium (NPE) and the outer Pigmented Epithelium (PE). (B) Comparison of the schematic diagram of the CE with actual histological section. (C) Eletron microscopy image of the CE indicating the PE and NPE ultrastructure.

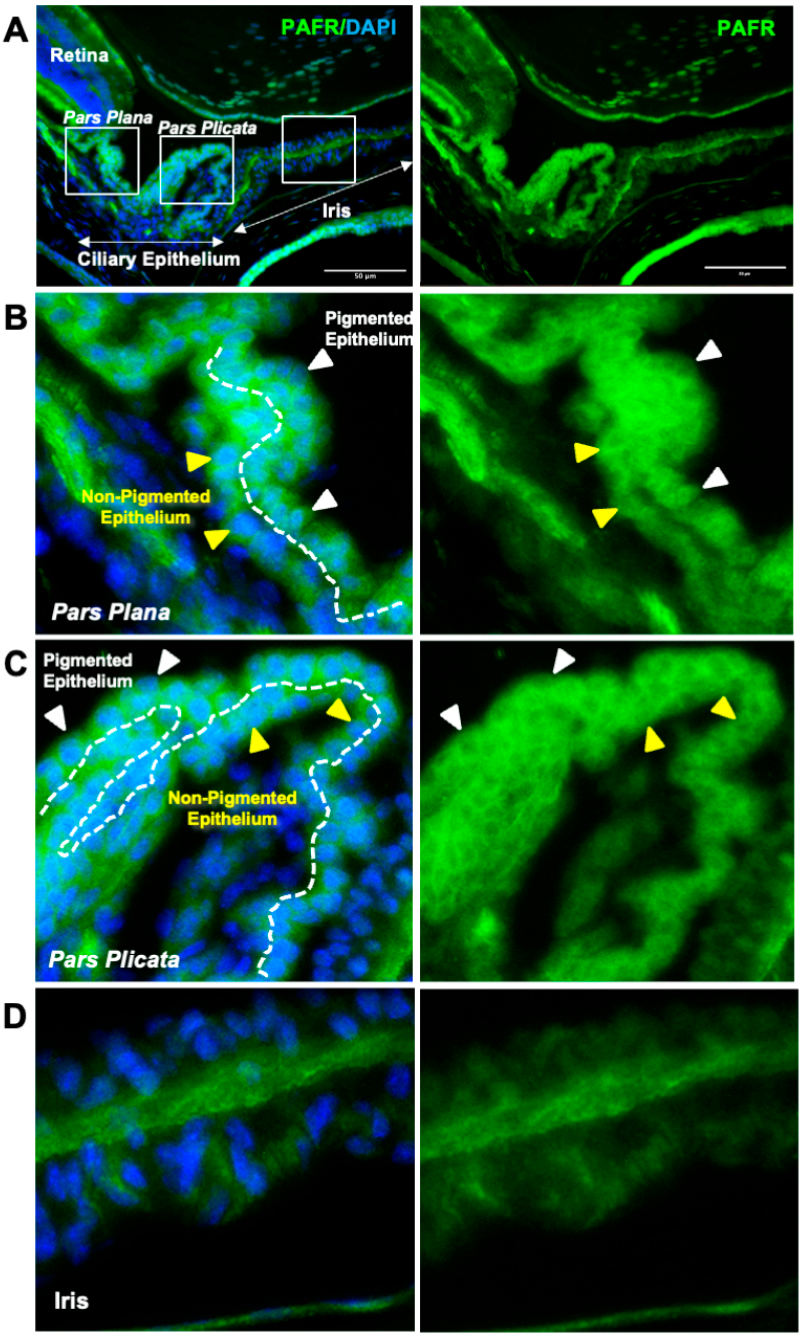

Figure S2. PAFR expression in CE cells. Immunofluorescence analysis of eyes' sections showing labeled cells for PAFR (green) and cell nucleus (DAPI) on IR, RE and CE (indicated by the double-head arrow) (n=3).

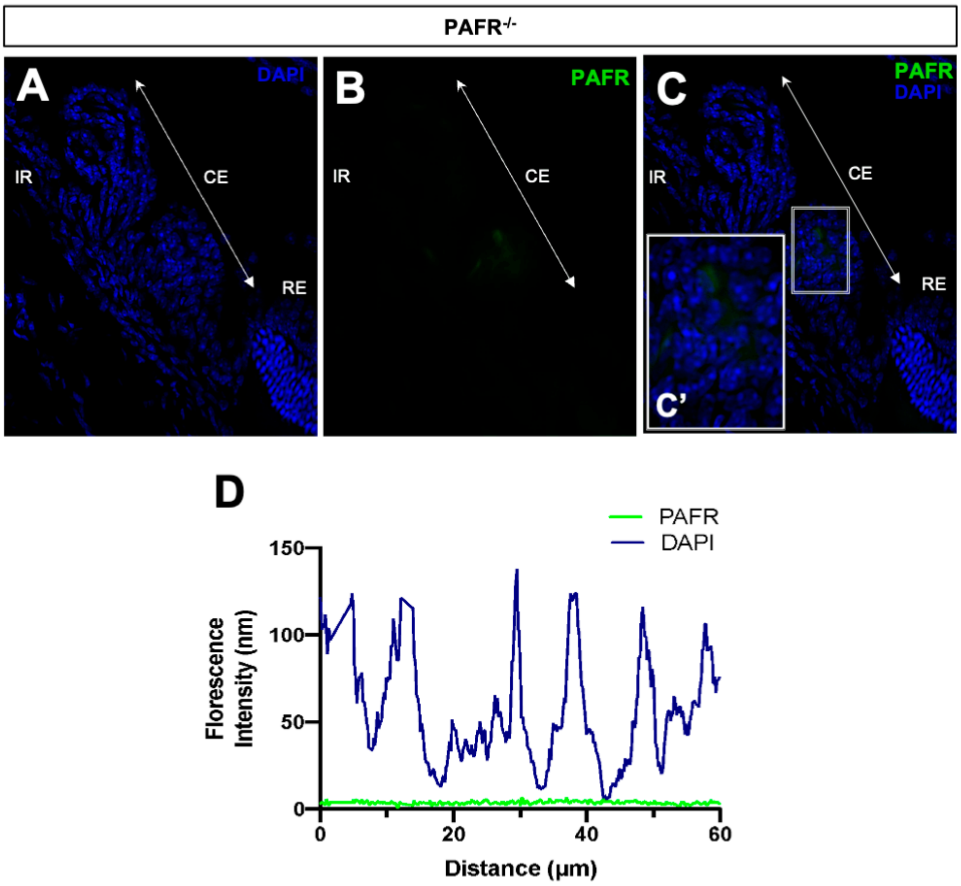

Figure S3. PAFR expression in PAFR-/- Ciliary Epithelium (CE). (A-C) Immunofluorescence analysis of CE section indicating the absence of PAFR expression. (green) and cell nuclei (DAPI, blue) in the iris (IR), retina (RE), and in the Ciliary Epithelium (CE). (n=3). (c') Detailed inset from figure C. (D) Confocal profile analysis on PAFR expression indicating the absence of green fluorescence

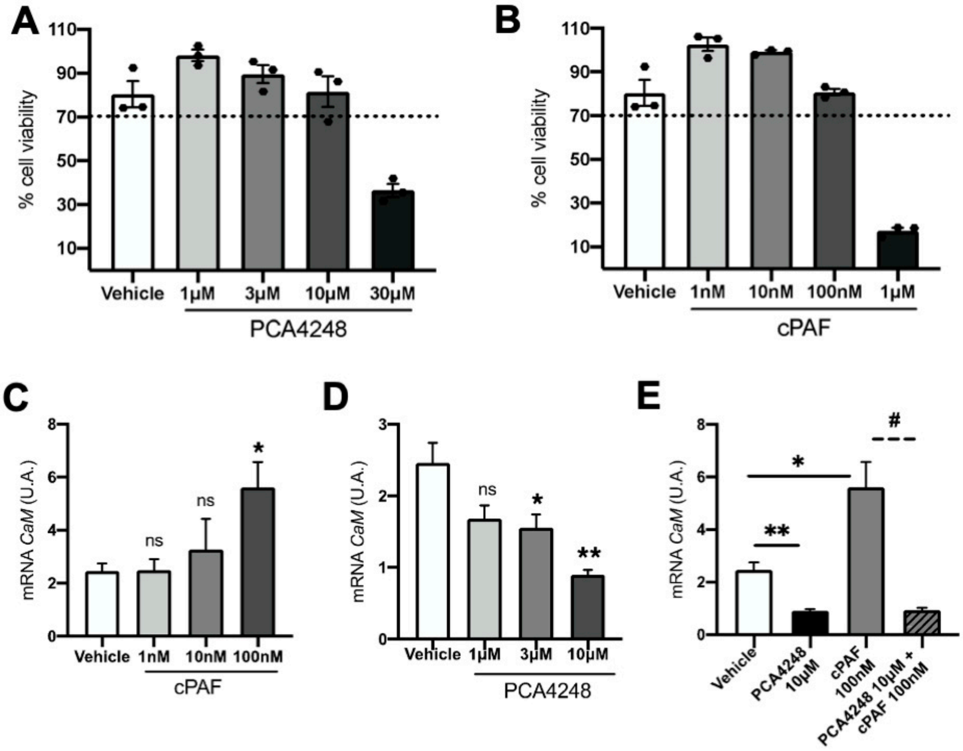

Figure S4. PAFR antagonist (PCA4248) and agonist (cPAF) viability and concentration test. PE cells were cultured for 48h with different concentrations of PAFR antagonist PCA4248 (1uM, 3uM, 10uM and 100uM) (A), or PAFR agonist cPAF (1nM, 10nM, 100nM and 1000nM) (B). Cell viability was determined by calcein assay. Treatment with viability above the 70% treshold were considered non-toxic (dotted line). Results are shown as mean ± SEM of comparative % of cell viability related to the vehicle group (1uM DMSO) (n=3). Transcriptional expression of Calmodulin (CaM) analyzed after 8h of treatment with non-toxic concentrations of PAFR antagonist PCA4248 (1uM, 3uM and 10uM) (C), PAFR agonist cPAF (1nM, 10nM, and 100nM) (D), and the combination of both selected concentrations (E) Results are given as mean ± SEM of comparative delta-CT related to vehicle group (1uM DMSO), * # P<0.05, ** P<0.01, ns = non-significant (n = 3), A.U. = Arbitrary Unit. PE = Pigmented Epithelium.

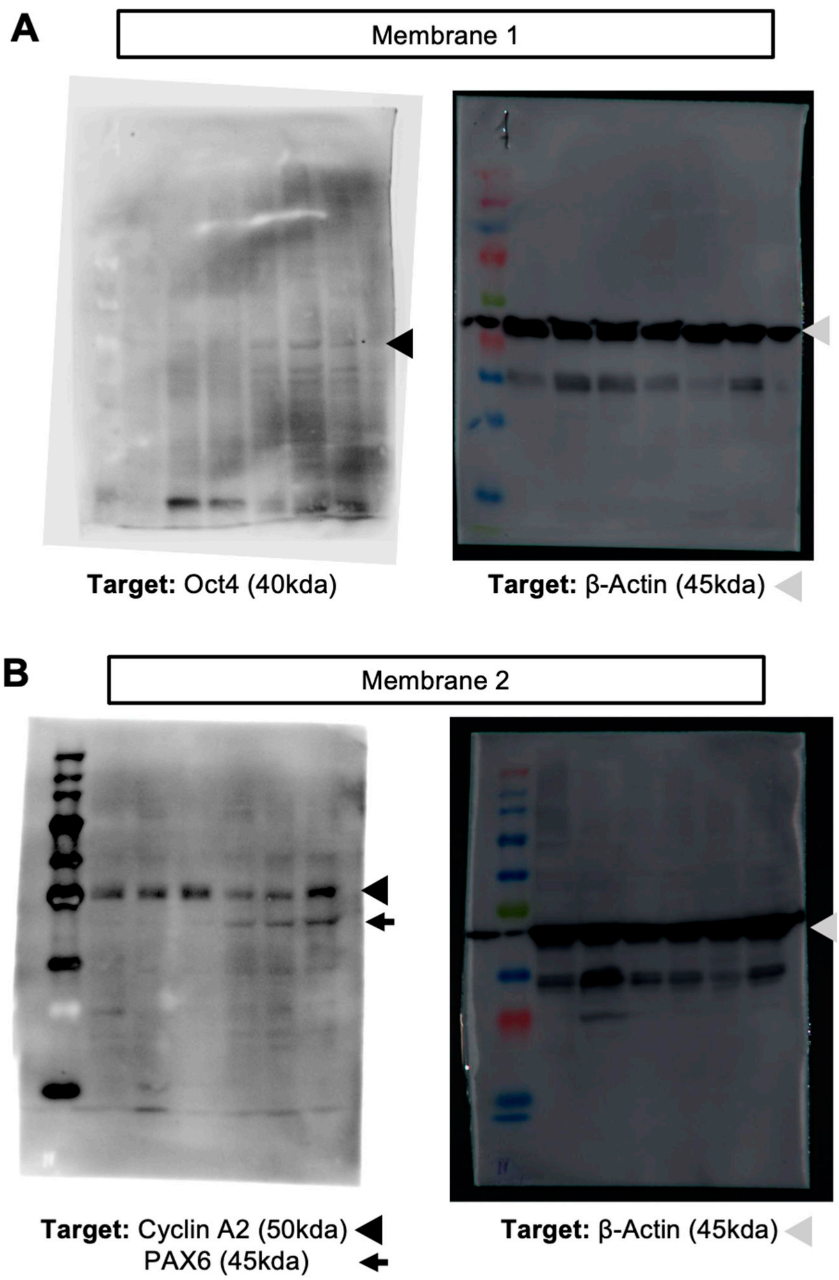

Figure S5. Western blot membranes of CE from WT and PAFR-/- animals. (A) Image of membrane 1 labeled with primary antibodies for Oct4 (40 kDa) (left) and β-Actin (45 kDa) (right). Bands 1-3 correspond to WT control animal samples, and bands 4-6 correspond to PAFR-/- animal samples. (B) Image of membrane 2 labeled with primary antibodies for Cyclin A2 (40 kDa) and PAX6 (45 kDa) (left) and β-Actin (45 kDa) (right). Bands 1-3 correspond to WT control animal samples, and bands 4-6 correspond to PAFR-/- animal samples.
